# Histone deacetylase SlHDA7 impacts fruit ripening and shelf life in tomato

**DOI:** 10.1093/hr/uhae234

**Published:** 2024-08-14

**Authors:** Yijie Zhou, Zhiwei Li, Xinguo Su, Huiyu Hou, Yueming Jiang, Xuewu Duan, Hongxia Qu, Guoxiang Jiang

**Affiliations:** State Key Laboratory of Plant Diversity and Specialty Crops & Guangdong Provincial Key Laboratory of Applied Botany, South China Botanical Garden, Chinese Academy of Sciences, Guangzhou 510650, China; Guangdong AIB Polytechnic, Guangzhou 510507, China; State Key Laboratory of Plant Diversity and Specialty Crops & Guangdong Provincial Key Laboratory of Applied Botany, South China Botanical Garden, Chinese Academy of Sciences, Guangzhou 510650, China; South China National Botanical Garden, Guangzhou 510650, China; University of Chinese Academy of Sciences, Beijing 100049, China; Guangdong AIB Polytechnic, Guangzhou 510507, China; State Key Laboratory of Plant Diversity and Specialty Crops & Guangdong Provincial Key Laboratory of Applied Botany, South China Botanical Garden, Chinese Academy of Sciences, Guangzhou 510650, China; South China National Botanical Garden, Guangzhou 510650, China; State Key Laboratory of Plant Diversity and Specialty Crops & Guangdong Provincial Key Laboratory of Applied Botany, South China Botanical Garden, Chinese Academy of Sciences, Guangzhou 510650, China; South China National Botanical Garden, Guangzhou 510650, China; University of Chinese Academy of Sciences, Beijing 100049, China; State Key Laboratory of Plant Diversity and Specialty Crops & Guangdong Provincial Key Laboratory of Applied Botany, South China Botanical Garden, Chinese Academy of Sciences, Guangzhou 510650, China; South China National Botanical Garden, Guangzhou 510650, China; University of Chinese Academy of Sciences, Beijing 100049, China; State Key Laboratory of Plant Diversity and Specialty Crops & Guangdong Provincial Key Laboratory of Applied Botany, South China Botanical Garden, Chinese Academy of Sciences, Guangzhou 510650, China; South China National Botanical Garden, Guangzhou 510650, China; University of Chinese Academy of Sciences, Beijing 100049, China; State Key Laboratory of Plant Diversity and Specialty Crops & Guangdong Provincial Key Laboratory of Applied Botany, South China Botanical Garden, Chinese Academy of Sciences, Guangzhou 510650, China; South China National Botanical Garden, Guangzhou 510650, China; University of Chinese Academy of Sciences, Beijing 100049, China

## Abstract

Fruit ripening depends on the accurate control of ripening-related genes expression, with histone deacetylases (HDACs) playing crucial roles in transcriptional regulation. However, the functions of HDACs in fruit maturation remain largely unexplored. Here, we show that SlHDA7 acts as a suppressor of fruit ripening and functions as an H4ac HDAC in tomato. Deletion of *SlHDA7* accelerated fruit ripening, while overexpression of *SlHDA7* delayed the maturation process. Additionally, ethylene production and carotenoid biosynthesis significantly increased in *slhda7* mutant fruits but decreased in *SlHDA7*-overexpressing fruits. Furthermore, SlHDA7 repress the expression of ethylene production and signaling, carotenoid metabolism, cell wall modification, and transcriptional regulation-related genes. RT-qPCR and ChIP-qPCR analyses indicated that SlHDA7 may deacetylate H4ac, leading to reduced transcript levels of *ACO1, GGPPS2*, *Z-ISO, EXP1*, and *XYL1* mRNA, consequently suppressing fruit ripening. Moreover, SlHDA7 suppresses fruit ripening by targeting specific ripening-associated transcription factors (TFs) like *RIN*, *FUL1*, and *ERF.E1*, ultimately leading to delayed ripening and prolonged fruit shelf life. In summary, our findings indicate that SlHDA7 negatively modulates tomato fruit maturation by adjusting H4ac levels of these ripening-associated genes and key TFs.

## Introduction

Fleshy fruit ripening, essential for fruit quality, is a complex genetically coordinated process. Increasing evidence indicates that fruit ripening is controlled by intricate networks that integrate internal and external factors, leading to significant alters in color, flavor, texture, and other attributes [1]. In the case of climacteric fruits like tomato, ethylene plays a vital role in regulating fruit maturation [2]. Subsequent studies indicate that ethylene-mediated regulation of fruit ripening depends on the accurate management of the expression of numerous ripening-associated genes, which are precisely regulated at the epigenetic and transcriptional levels [3, 4].

Epigenetic modifications, such as DNA methylation, noncoding RNA, and histone modifications (methylation, acetylation, ubiquitination, phosphorylation, and so on), act as a crucial regulatory mechanism that controls chromatin status and gene transcription [5–7]. DNA methylation/demethylation is vital for fruit ripening [8–11]. Knocking out DNA demethylase (*SlDML2)* causes a non-ripening phenotype in tomato, indicating that SlDML2 plays a crucial role in regulating tomato fruit maturation [9, 12]. Notably, *SlDML2*-knockout fruits exhibit defective ethylene production, highlighting the importance of SlDML2 in ripening-induced ethylene biosynthesis [8, 12]. Alongside DNA methylation/demethylation, reversible methylation/demethylation of histones appears to be a critical mechanism for regulating ripening-associated gene expression and is essential for tomato fruit ripening [13, 14]. Despite the significant roles of epigenetic modifications in fruit maturation, the molecular regulation of these ripening-related genes by epigenetic modifications still requires further elucidation.

Histone acetylation, another conserved histone modification, plays vital roles in controlling chromatin structure and gene expression [15]. Histone deacetylases (HDACs) are highly conserved enzymes in eukaryotes and are responsible for removing acetyl groups from acetylated histones at lysine residues [15–17]. Over the past decade, HDACs have garnered significant attention and have been demonstrated to play crucial roles in the plant development process, including seed [18], vegetative development [19, 20], root epidermis [21, 22], flower development [23, 24], and leaf senescence [25], as well as stress responses [26]. In fruits, HDACs have been implicated as crucial regulators of fruit development and ripening [27–31]. For instance, CsHDC1 regulates cytokinin and polyamine biosynthesis and metabolism, and its downregulation results in a short-fruit phenotype [31]. MaHDA1 and CpHDA3 have been shown to suppress fruit ripening by directly interacting with the transcriptional repressors MaERF11 and CpERF9, respectively [ 27]. Similarly, MdHDA19 inhibits apple fruit ripening via interacting with MdERF4-MdTPL4 to directly repress ripening-related gene expression [32]. In tomato, silencing of *SlHDA1*, *SlHDA3*, or *SlHDT1* enhances fruit ripening, while silencing of *SlHDT3* hinders fruit ripening [28, 29, 33–35]. Particularly noteworthy, SlERF.F12 directly interacts with the corepressor SlTPL2 and recruits SlHDA1/SlHDA3 to create a transcriptional complex, leading to the suppression of the initiation of tomato fruit maturation by repressing numerous ripening-associated genes’ expression [34]. Despite numerous reported roles of HDACs in regulating fruit maturation, the underlying mechanism of HDACs in fruit maturation remain largely unclear.

Here, we illustrated the function of the RPD3/HDA1 subfamily gene *SlHDA7* in controlling fruit ripening in tomato. Deletion of *SlHDA7* accelerated fruit ripening, while overexpression of *SlHDA7* hindered fruit ripening. Through molecular and biochemical analysis, we showed that *SlHDA7* suppresses the expression of ripening-associated genes by reducing histone acetylation levels, leading to decreased ethylene production and carotenoid biosynthesis during tomato fruit ripening. This research offers detailed insights into the function of *SlHDA7* in fruit maturation and unveils a new understanding of the underlying mechanism of fruit maturation and carotenoid biosynthesis.

## Results

### Expression profile of *SlHDA7* during tomato fruit maturation

**Figure 1:**
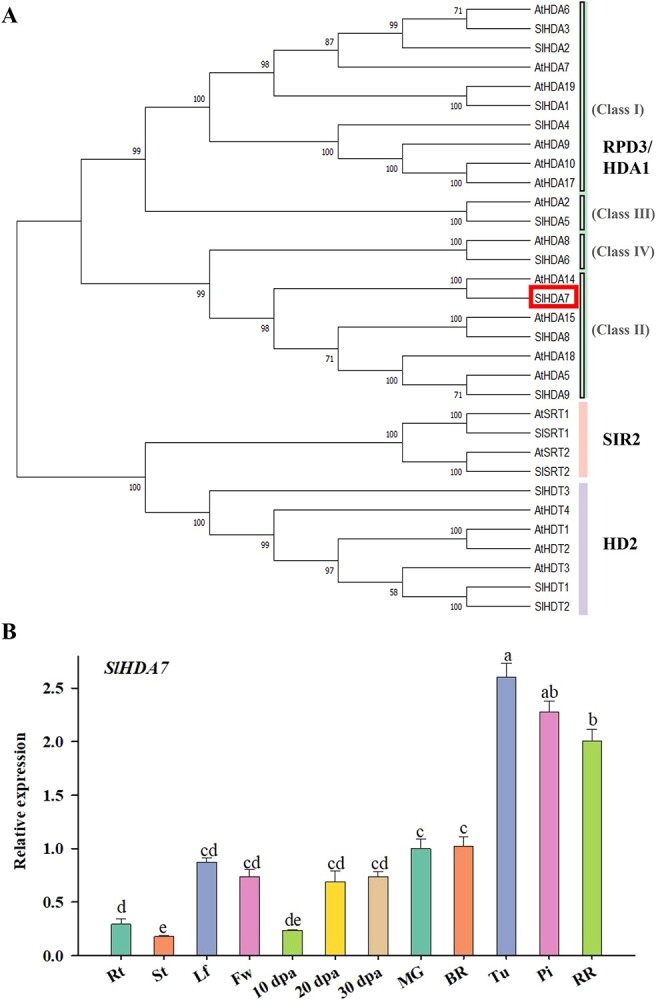
Phylogenetic analysis and expression profiles of *SlHDA7*. **A** Phylogenetic analysis of histone deacetylases (HDACs) from Arabidopsis and tomato. **B** Transcript levels of *SlHDA7* in different tissues and at various developmental stages of tomato. BR, break stage; dpa, days post anthesis; Fw, flower; Lf, leaf; MG, mature green stage; Pi, pink stage; RR, red ripe stage; Rt, root; St, stem; Tu, turning stage. Data are means ± SD (*n* = 3). Different letters above bars indicate significant differences compared to fruit at MG stage by Student’s *t* test (*P < 0.05).

Previous study has shown that the HD2 and subclass I members of RPD3/HDA1 family HDACs, including SlHDA1, SlHDA3, SlHDT1, and SlHDT3, play important roles in fruit ripening by regulating ethylene biosynthesis and carotenoid metabolism [28, 29, 33, 35, 36]. However, the function of the subclass II clade HDAC remains unexplored. According to a previous study [37], the subclass II clade HDAC genes *SlHDA7* and *SlHDA10* exhibited a ripening-induced expression pattern, with a particular emphasis on SlHDA7. Hence, we focused our further study on SlHDA7. SlHDA7 has an open reading frame (ORF) spanning 798 base pairs (bp), encodes a 265 amino acid protein with active sites similar to other plant HDACs and an isoelectric point predicted at 5.89. Alignment of amino acid sequence and phylogenetic analysis revealed that SlHDA7 was highly homologous to AtHDA14 (Fig. 1A), which has been implicated in regulating photosynthesis and melatonin biosynthesis [38–40].

**Table 1:** Ripening-associated genes expression changes were compared between *slhda7–1* mutant and WT fruit at 36 dpa.

**Gene name**	**Gene ID**	**Log2(fold change)**	**Annotation**
**Ethylene biosynthesis and signaling**			
*ACS2*	Solyc01g095080	4.533	1-aminocyclopropane-1-carboxylate synthase 2
*ACS4*	Solyc05g050010	3.533	1-aminocyclopropane 1-carboxylate synthase 4
*ACO1*	Solyc07g049530	3.065	1-aminocyclopropane-1-carboxylate oxidase 1
*ACO3*	Solyc07g049550	1.598	1-aminocyclopropane-1-carboxylate oxidase 3
*ETR3*	Solyc09g075440	1.591	Ethylene receptor never ripe
*ETR4*	Solyc06g053710	1.381	Ethylene receptor homolog precursor
*ERF.E1*	Solyc09g075420	2.457	Ethylene response factor 1
*ERF.E3*	Solyc03g123500	1.069	Ethylene response factor 3
*ERF.E4*	Solyc01g065980	1.010	Ethylene response factor 4
*ERF.H1*	Solyc06g065820	4.285	Ethylene response factor 3-like
**Carotenoid biosynthesis**			
*DXS1*	Solyc01g067890	1.821	1-D-deoxyxylulose 5-phosphate synthase
*IDI1*	Solyc04g056390	1.103	Isopentenyl diphosphate isomerase
*GGPPS1*	Solyc11g011240	2.534	Geranylgeranyl pyrophosphate synthase 1
*GGPPS2*	Solyc04g079960	5.113	Geranylgeranyl pyrophosphate synthase 2
*PSY1*	Solyc03g031860	3.969	Phytoene synthase 1
*CrtISO*	Solyc10g081650	1.728	Prolycopene isomerase
*ZISO*	Solyc12g098710	5.890	15-cis-zeta-carotene isomerase
*CrtR-b2*	Solyc03g007960	1.639	Beta-carotene hydroxylase
**Cell wall degradation**			
MAN4	Solyc01g008710	6.567	Mannan endo-1,4-beta-mannosidase 4 precursor
*PL*	Solyc03g111690	2.764	Pectate lyase 18
*PME1*	Solyc03g123620	1.065	Pectinesterase
*EXP1*	Solyc06g051800	2.574	Expansin precursor 1
*CEL8*	Solyc08g082250	1.643	Endo-beta-1,4-D-glucanase precursor 8
*CEL2*	Solyc09g010210	3.678	Endo-1,4-beta-glucanase precursor 2
*PL8*	Solyc09g091430	6.352	PREDICTED: probable pectate lyase 8
*XYL1*	Solyc10g047030	3.010	Beta-D-xylosidase 1 precursor
*PG2a*	Solyc10g080210	8.518	Polygalacturonase-2 precursor
*TBG4*	Solyc12g008840	4.448	Galactosidase precursor
**Maturation regulator and chlorophyll metabolism**			
*AP2a*	Solyc03g044300	1.165	APETALA2-like protein
*NAC9*	Solyc04g005610	1.582	PREDICTED: NAC transcription factor 29
*RIN*	Solyc05g012020	3.712	MADS-box transcription factor isoform 1
*FUL1*	Solyc06g069430	2.697	AGAMOUS-like MADS-box protein AGL8 homolog
*E4*	Solyc03g111720	4.582	Methionine sulfoxide reductase
*E8*	Solyc09g089580	4.477	1-aminocyclopropane-1-carboxylate oxidase -like
*SGR1*	Solyc08g080090	1.714	Senescence-inducible chloroplast stay-green protein 1
*CAO*	Solyc11g012850	1.279	Chlorophyllide a oxygenase

To further analyse the possible role of SlHDA7 in tomato plants, we examined its expression pattern in various tissues and fruit pericarps across development and ripening stages using RT-qPCR. Our findings revealed significantly higher expression levels of *SlHDA7* in fruits and leaves compared to stems and roots (Fig. 1B). Moreover, its expression in fruits experienced a substantial upregulation during early ripening, reaching a peak at the turning stage, and then decreased in the late ripening phase (Fig. 1B), similar to previously published RNA-seq data (Fig. S1, see online supplementary material). This is slightly different from the previous report [37], which may be due to variations in the varieties. These results suggest that SlHDA7 might play a regulatory role in tomato fruit maturation.

### SlHDA7 suppresses tomato fruit maturation

**Figure 2:**
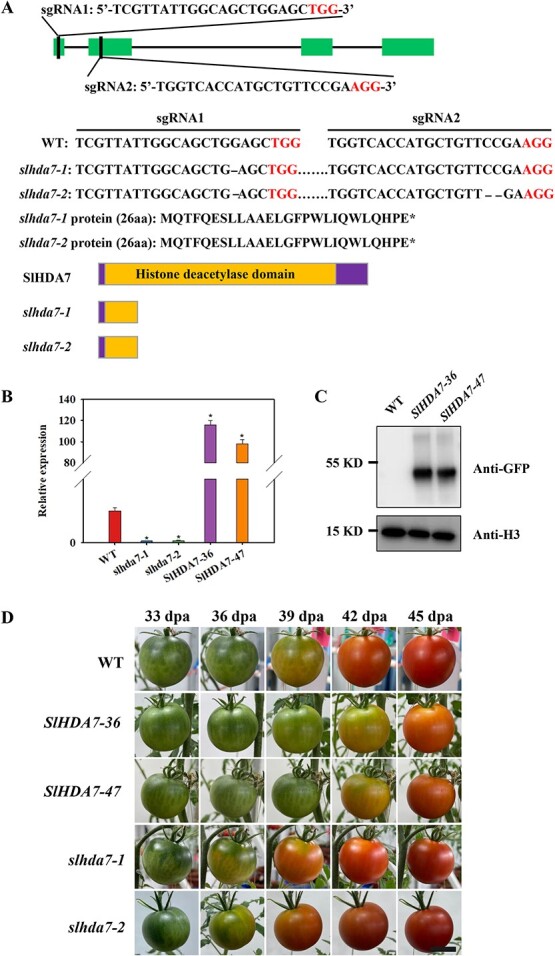
SlHDA7 represses tomato fruit maturation. **A** Generation of *SlHDA7* knockout mutants. **B** Analysis of the gene expression level of *SlHDA7* in the *SlHDA7* overexpression and knockout mutant lines by RT-qPCR. *ACTIN* was used as the internal control. Asterisks indicate significant differences compared to WT by Student’s *t* test (^*^P < 0.05). **C** Western blot analysis of the protein level of SlHDA7 in the *SlHDA7* overexpression plants. An anti-GFP antibody was used for detection, and an anti-H3 antibody was used for internal control. **D** Phenotypes of the WT, *SlHDA7* overexpression lines *SlHDA7–36* and *SlHDA7–47*, and the *SlHDA7* knockout mutant lines *slhda7–1* and *slhda7–2* during fruit maturation. Bar = 2 cm.

To explore the impact of altered *SlHDA7* expression on fruit maturation, we utilized the CRISPR/Cas9 gene editing system to develop *SlHDA7* knockout mutants. Two *slhda7* mutants, *slhda7–1* and *slhda7–2,* were isolated with a homozygous 1-bp deletion in the first exon, resulting in a predicted 3.1 kD truncated protein (Fig. 2A). *slhda7–2* mutant also carried an addition 2-bp deletion caused by target sgRNA2 (Fig. 2A). The *SlHDA7* transcript was barely detectable in these mutant lines (Fig. 2B). Additionally, we created stable transgenic plants with increased *SlHDA7* levels*.* Two distinct transgenic lines, *SlHDA7–36* and *SlHDA7–47*, with a C-terminal GFP fusion, showed 115- and 97-fold higher expression levels of *SlHDA7* compared to the wild type (WT), and were chosen for further examination (Fig. 2B and C).

**Figure 3:**
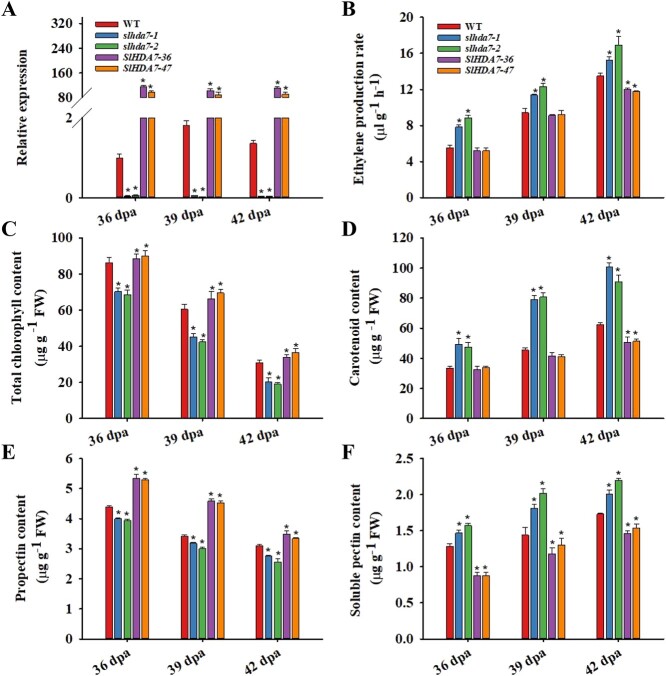
Expression level of *SlHDA7* and ripening-related physiological properties of the WT, *SlHDA7-OE*, and *slhda7* mutant at 36, 39, and 42 dpa. **A** Relative expression levels of *SlHDA7* in the *slhda7–1*, *slhda7–2*, *SlHDA7–36*, and *SlHDA7–47* lines by RT-qPCR. *ACTIN* was used as the reference control. **B**–**F** Ethylene production rate (**B**), total chlorophyll content (**C**), carotenoid content (D), propectin content (**E**), and soluble pectin content (**F**) in the fruits of the WT, *SlHDA7-OE* lines, and *slhda7* mutant fruits at 36, 39, and 42 dpa. Data are means ± SD (*n* = 3). Asterisks indicate significant differences compared to WT by Student’s *t* test (^*^P < 0.05).

Phenotypic analysis indicated that the *SlHDA7-OE* lines exhibited delayed fruit maturation compared to WT fruits, while the absence of *SlHDA7* function led to early ripening (Fig. 2D). Corresponding to the ripening phenotype, *slhda7–1* and *slhda7–2* fruits displayed higher ethylene production rates, along with increased carotenoid and soluble pectin contents, but lower chlorophyll and protopectin levels compared to WT fruits. Conversely, the *SlHDA7-OE* fruits demonstrated contrasting patterns for these physiological characteristics (Fig. 3B–F). These findings indicate that SlHDA7 functions as a suppressor of tomato fruit maturation.

### SlHDA7 deacetylates H4ac in tomato

**Figure 4:**
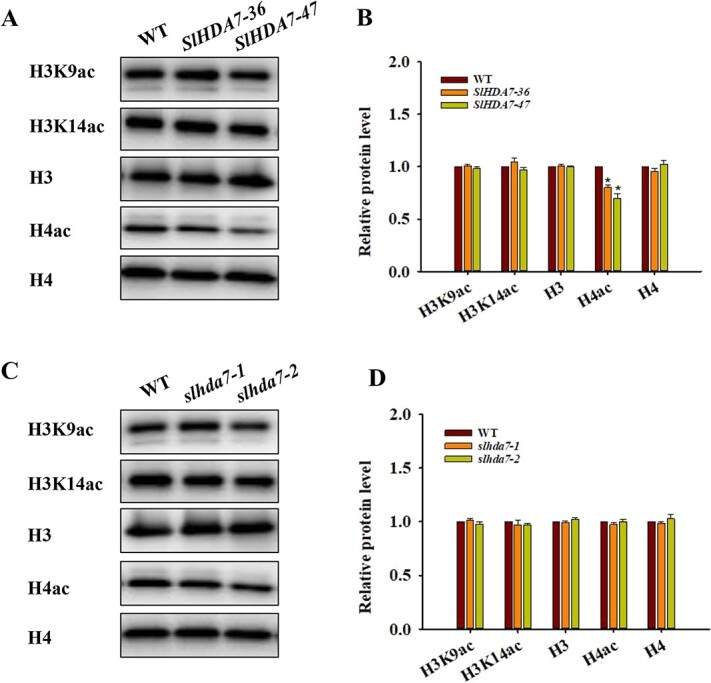
SlHDA7 functions as a histone H4ac deacetylase. **A** Assessment of global acetylation levels of H3K9, H3K14, and H4 in the WT and *SlHDA7-OE* lines. **B** Statistical evaluation of Fig. 4A. **C** Assessment of global acetylation levels of H3K9, H3K14, and H4 in the WT and *slhda7* mutant lines. **D** Statistical evaluation of Fig. 4C. The H3 and H4 were utilized as a loading controls. Data are means ± SE (*n* = 3). Asterisks indicate significant differences compared to WT by Student’s *t* test (^*^P < 0.05).

Given the correlation between HDACs function and their deacetylase activity, we verified SlHDA7’s histone deacetylation activity by comparing histone lysine acetylation levels in the WT, *slhda7* mutant, and *SlHDA7-OE* lines using specific antibodies. The H4ac levels in the *SlHDA7-OE* lines were lower than these in the WT (Fig. 4A and B), while there were no significant differences in H4ac levels in the *slhda7* mutants (Fig. 4C and D). Additionally, global levels of H3K9ac and H3K14ac showed no significant variance among the WT, *slhda7* mutants, and *SlHDA7-OE* lines (Fig. 4A–D). These findings suggest that SlHDA7 may be responsible for H4ac deacetylation during tomato fruit ripening.

### SlHDA7 regulates the expression of ripening-related genes

**Figure 5:**
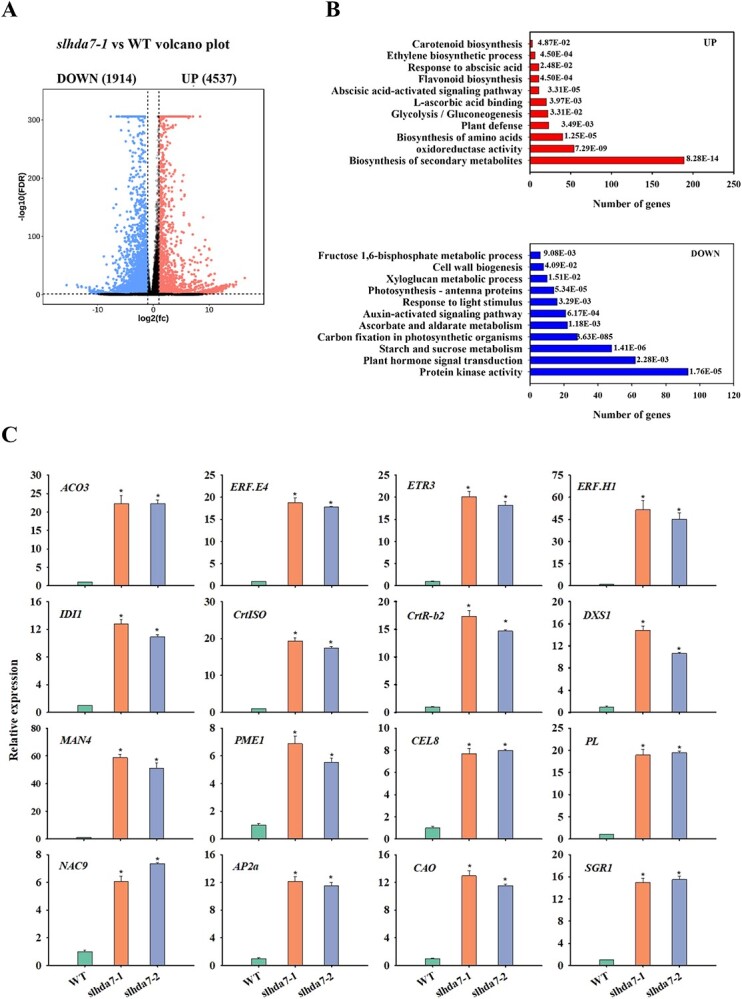
Transcriptome analysis of DEGs modulated by *SlHDA7* in tomato fruits. **A** Volcano plots of the RNA-seq data. **B** GO enrichment analysis of SlHDA7-upregulated and downregulated genes by DAVID. **C** Validation the results of RNA-seq by RT-qPCR. *ACTIN* was used as the reference control. Data are means ± SD (*n* = 3). Asterisks indicate significant differences compared to WT by Student’s *t* test (^*^P < 0.05).

To identify genes influenced by SlHDA7 during tomato fruit ripening, we compared the transcriptomes of WT and *slhda7–1* fruits at 36 dpa by RNA-seq analysis. A total of 6451 genes exhibited significant differential expression (fold change >2.5, FDR < 0.05) between WT and *slhda7–1* fruits. Among the 6451 genes regulated by SlHDA7, 1914 were suppressed, and 4537 were activated (Fig. 5A; Table S2, see online supplementary material). Gene Ontology (GO) enrichment analysis indicated that the upregulated genes in *slhda7–1* fruits were notably involved in secondary metabolite biosynthesis and oxidoreductase activity, whereas the downregulated genes were associated with protein kinase activity and plant hormone signal transduction pathways (Fig. 5B). Moreover, numerous genes associated with ripening-related transcription regulation, the abscisic acid-activated signalling pathway, the ethylene biosynthesis process, flavonoid biosynthesis, and carotenoid biosynthesis were notably upregulated in the *slhda7–1* fruits (Fig. 5B; Table 1; Table S2, see online supplementary material).

To clarify whether *SlHDA7* indirectly regulates tomato fruit ripening through other HDACs, we analysed the expression of previously reported *SlHDACs* in RNA-seq data. The result revealed that the expression of *SlHDACs* did not show significant difference between the WT and the *slhda7* mutant fruits except for *SlHDA7*, *SlHDA5*, and *SlHDT3* (Fig. S2, see online supplementary material). Observing this, we first consider whether the sequences similarity among HDACs results in CRISPR/Cas9 off target effects, leading to reduced expression of *SlHDA5* and *SlHDT3*. However, a multiple sequence alignment of sgRNA1 and sgRNA2 among tomato *SlHDACs* revealed that both sgRNAs were specific to *SlHDA7* and did not cause off-target effects (Fig. S3, see online supplementary material). Additionally, *SlHDT3* is known to positively regulate tomato fruit ripening [35], while *SlHDA5* responds to abiotic stress [41]. Because the expression of *SlHDT3* is significantly downregulated during tomato fruit ripening, particularly from IMG to BR stage [35, 37], the unexpected down-regulation expression of *SlHDT3* in *slhda7* mutant may be related to the WT fruit at the mature green stage, whereas the *slhda7* fruit is at the break stage. SlDML2 mediates DNA demethylation of ripening-related genes, playing a crucial regulatory role in tomato fruit ripening. Our study revealed that the expression of *SlDML2* did not show significant difference between the WT and the *slhda7–1* fruits. Moreover, DNA methylation in the *SlHDA7* promoter did not differ significantly between WT and the *sldml2* fruits [12]. Taken together, these findings indicated that the ripening changes in the *slhda7* fruits may primarily due to the absence of *SlHDA7.*

In addition, we conducted an RT-qPCR assay to confirm the accuracy of the RNA-seq results. The expression of the 16 fruit ripening-associated genes, such as *ACO3*, *ERF.E4*, *ETR3*, *ERF.H1*, *IDI1*, *CrtISO*, *CrtR-b2*, *DXS1*, *MAN4*, *PME1*, *CEL8*, *PL*, *NAC9*, *AP2a*, *CAO*, and *SGR1* were confirmed to be upregulated in the *slhda7* fruits compared with the WT fruits (Fig. 5C). Overall, these findings indicate that SlHDA7 delays fruit ripening by influencing the expression of ripening-related genes associated with ethylene biosynthesis and response, carotenoid metabolism, cell wall modification, and transcriptional regulation.

### SlHDA7 inhibits the expression of ripening-related genes via deacetylation of H4ac during tomato fruit maturation

**Figure 6:**
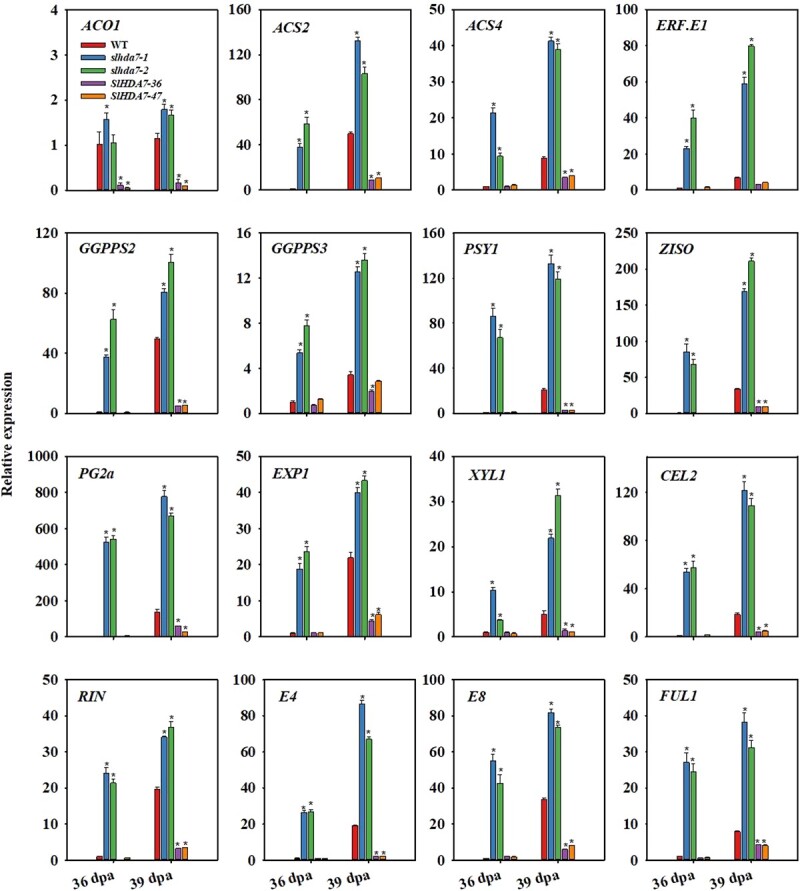
Ripening-associated genes expression profiles in WT, *slhda7*, and *SlHDA7-OE* fruits at 36 and 39 dpa. *ACTIN* was used as the reference control. Data are means ± SD (*n* = 3). Asterisks indicate significant differences compared to WT by Student’s *t* test (^*^P < 0.05).

Previous studies have revealed that fruit ripening is dependent on the precise regulation of numerous ripening-related genes, which are tightly controlled at the epigenetic and transcriptional levels [3, 4]. Based on our previous findings, we postulated that SlHDA7 retards fruit ripening through its H4ac deacetylase activity. To validate this hypothesis, we initially investigated the expression of key maturation-related genes, such as *ACO1*, *ACS2*, *ACS4*, *ERF.E1*, *GGPPS2*, *GGPPS3*, *PSY1*, *ZISO*, *PG2a*, *EXP1*, *XYL1*, *CEL2*, *RIN*, *E4*, *E8*, and *FUL1* in WT, *SlHDA7-OE,* and *slhda7* mutant fruits. As expected, RT-qPCR analysis revealed up-regulation of these genes in *slhda7* mutant fruits, while down-regulation in *SlHDA7-OE* fruits at 36 and 39 dpa (Fig. 6).

**Figure 7:**
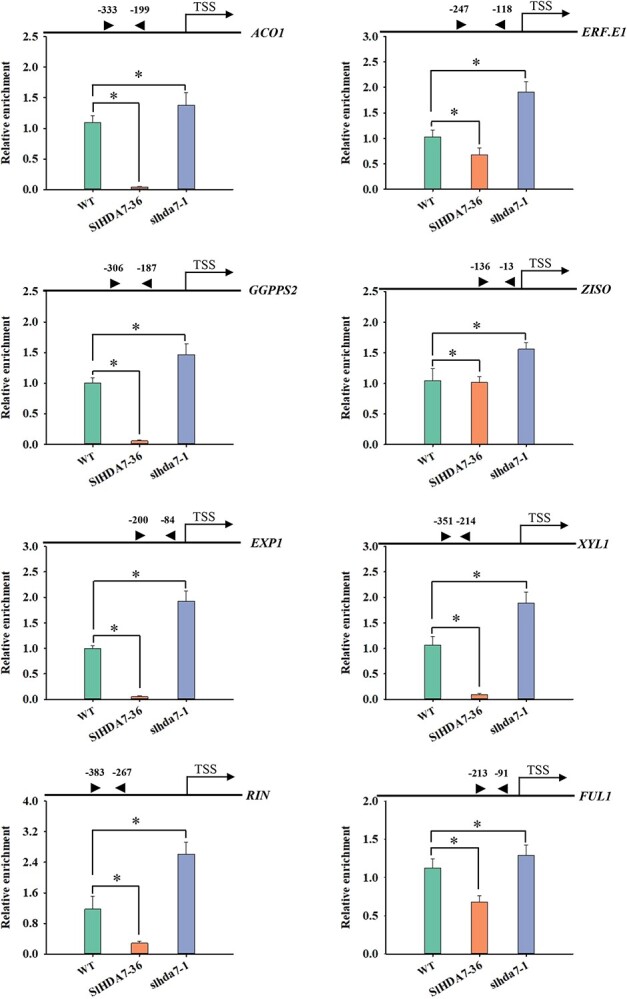
ChIP-qPCR analysis of the H4ac levels of ripening-associated genes in *SlHDA7-OE*, *slhda7*, and WT fruits. The black arrows indicate the positions of the different primers used for ChIP-qPCR. Chromatin was isolated from the WT, *slhda7–1*, and *SlHDA7–36* fruits at 36 dpa with an anti-H4ac antibody. *ACTIN* was used as the reference control. Data are means ± SD (*n* = 3). Asterisks indicate significant differences compared to WT by Student’s *t* test (^*^P < 0.05).

To further validate this hypothesis, we conducted ChIP-qPCR assays to evaluate the H4ac levels of these ripening-related genes in *SlHDA7-OE*, *slhda7*, and WT fruits at 36 dpa. In comparison to WT fruits, the H4ac levels at the promoter regions of *ACO1*, *ERF.E1*, *GGPPS2*, *ZISO*, *EXP1*, *XYL1*, *RIN*, and *FUL1* were decreased in *SlHDA7-OE* fruits but notably increased in *slhda7* fruits (Fig. 7). Because H4ac is an active marker, these results suggest that SlHDA7 might function as a suppressor of fruit maturation by inhibiting the transcription of certain ripening-related genes through the elimination of H4ac.

### Knockout *SlHDA7* shortens the fruit shelf life

**Figure 8:**
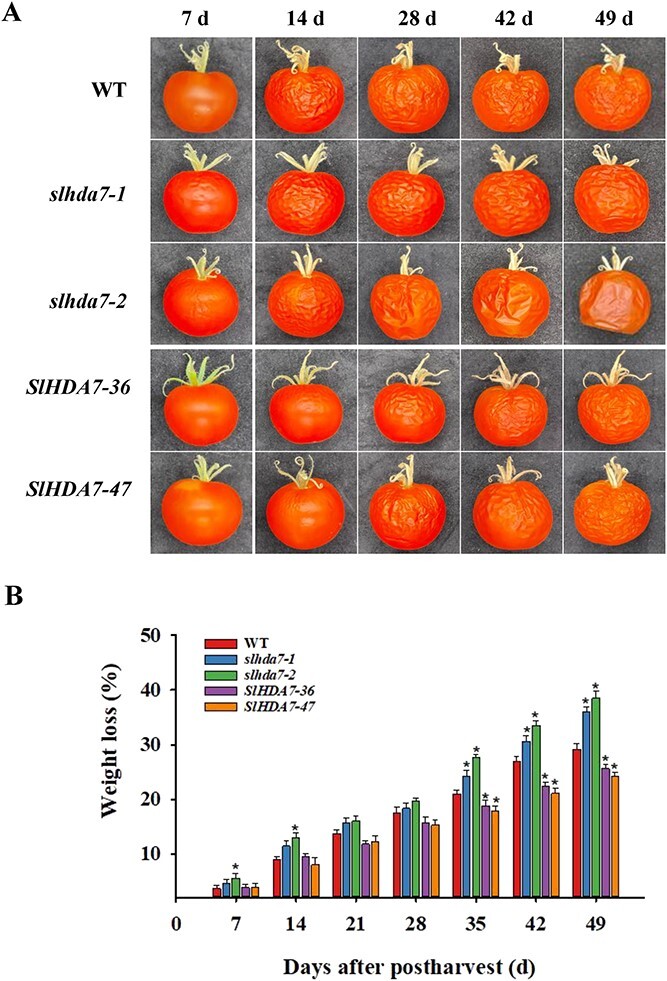
SlHDA7 extends the fruit shelf life of tomato. **A** Phenotypes of WT, *SlHDA7-OE*, and *slhda7* mutant fruits stored for 49 days. **B** The weight loss rate of WT, *SlHDA7-OE*, and *slhda7* mutant fruits during 49 days post-harvest storage. Data are means ± SD (*n* = 3). Asterisks indicate significant differences compared to WT by Student’s *t* test (^*^P < 0.05).

To investigate the impact of *SlHDA7* overexpression and knockout on the postharvest shelf life, fruits of WT, *SlHDA7-OE*, and *slhda7* were harvested at the Br + 7 stage (the 7th day after the break stage) and stored under the same conditions at room temperature. The skin of the WT fruits began to wrinkle after 7 days of storage, while the *SlHDA7-OE* fruits only showed slight shrinkage after 14 days of storage (Fig. 8A). In contrast, the *slhda7* fruits experienced accelerated shrinkage, with severe shrinkage and deformation after 49 days of storage (Fig. 8A). To explore the potential mechanism linked to the fruit shelf life of the WT, *SlHDA7-OE*, and *slhda7* fruits, we monitored the weight loss rate during storage. As depicted in Fig. 8B, the weight loss rate of the *slhda7* fruits was notably higher than that of WT fruits after 35 days of storage, but significantly lower for *SlHDA7-OE* fruits (Fig. 8B). These findings suggest that *SlHDA7* prolongs fruit shelf life by reducing water loss rate and delaying ripening.

## Discussion

Fruit ripening, the final stage of fruit development, is synergistically controlled by both internal developmental factors and external environmental factors. It depends on the accurate control of numerous ripening-related genes to remodel biochemical and physiological processes that determine fruit quality [42]. Despite the identification of numerous epigenetic factors associated with fruit ripening [14, 34, 36], the functions and impacts of most HDACs in controlling this process remain ambiguous. In this study, we demonstrate that SlHDA7, a subclass II clade histone deacetylase, might function as a suppressor of tomato fruit maturation by deacetylating H4ac to inhibit the transcription of crucial ripening-related genes.

### SlHDA7 functions as a suppressor of tomato fruit maturation

Histone acetylation is an essential epigenetic marker closely linked to gene activation or inhibition. Recent research indicates that histone acetylation and deacetylation, regulated by histone acetyltransferases and histone deacetylases, play crucial functions in plant development, stress response, and fruit ripening [13, 36, 43]. For instance, AtHDA9 depressingly affects salt and drought stress by modulating histone acetylation levels of numerous stress-responsive genes in Arabidopsis [44]. MaHDA1 and MdHDA19 negatively regulate banana fruit ripening via directly interacting with transcriptional repressors to form a repressor complex to suppresses the expression of many ripening-related genes [27, 32]. In tomato, *SlHDA1*, *SlHDA3*, or *SlHDT1* delay fruit ripening, while *SlHDT3* promotes it [28, 29, 33, 35]. Despite the reported essential roles of HDACs in modulating fruit maturation, the regulatory mechanisms of HDACs in this process remain largely unclear.

Here, we present evidence supporting that SlHDA7 functions as a suppressor of fruit maturation in tomato. Firstly, the expression of *SlHDA7* significantly increased during tomato fruit maturation (Fig. 1C). Additionally, knockout of *SlHDA7* was found to accelerate tomato fruit ripening, whereas overexpression of *SlHDA7* led to delayed ripening (Fig. 2D). Moreover, a multitude of ripening-associated genes participated in ethylene production, carotenoid synthesis, cell wall metabolism, and key ripening-related transcription factors were upregulated in the *slhda7* mutant fruits, while downregulated in *SlHDA7* overexpression fruits (Fig. 6). These findings collectively suggest that SlHDA7 exerts a negative influence on tomato fruit ripening.

### SlHDA7 inhibits tomato fruit maturation by removing H4ac at ripening-related gene loci

In eukaryotes, histone acetylation and deacetylation are directly connected with chromatin status and genes expression, while HDACs control gene expression via removing acetyl groups from histone tails, resulting in chromatin condensation and transcriptional repression. Our RNA-seq data revealed that *SlHDA7* likely modulate the expression of various maturation-related genes participating in ethylene production and signaling, carotenoid synthesis, cell wall metabolism, and ripening-related transcription factors, underscoring the significance of SlHDA7 in coordinating tomato fruit ripening transcription (Fig. 5). To uncover the function of SlHDA7 in tomato fruit maturation, we performed the RT-qPCR and ChIP-qPCR assays to identify putative target genes of SlHDA7.

Numerous studies have revealed that ethylene is the main trigger of climacteric fruit ripening [2, 45, 46]_._ In the present study, knockout of *SlHDA7* promoted ethylene biosynthesis, whereas overexpression of *SlHDA7* suppressed this process (Fig. 3C). Furthermore, key genes involved in ethylene synthesis and signaling, such as *ACO1*, *ACS2*, *ACS4*, and *ERF.E1* [46, 47] were significantly upregulated in *slhda7* fruits but downregulated in *SlHDA7-OE* fruits (Fig. 6). Additionally, ChIP-qPCR assays indicated that *SlHDA7* suppresses the expression of *ACO1* and *ERF.E1* by reducing H4ac levels at their gene loci during tomato fruit ripening (Fig. 7). Carotenoid accumulation and cell wall degradation lead to the red softened fruit, which are the two major characteristics of tomato ripening. Our findings revealed that SlHDA7-mediated removal of H4ac represses the expression of crucial carotenoid biosynthesis (*GGPPS2* and *ZISO*) and cell wall metabolism (*EXP1*and *XYL1*) related genes (Fig. 7). Collectively, these findings indicate that *SlHDA7* delays tomato fruit ripening via inhibiting ripening-related structural genes expression.

In addition to ripening-related structural genes, transcription factors orchestrate fruit ripening via the integration of internal signals with environmental cues [48, 49]. In tomato, *RIN* and *FUL1* are crucial for fruit ripening based on mutant or knockout analyses [50, 51]. Notably, *RIN* and *FUL1* positively regulate the tomato fruit maturation by controlling various maturation-related genes participating in ethylene synthesis and signaling, chlorophyll degradation, carotenoid metabolism, and cell wall degradation, and transcription regulation [52–54]. In this work, we revealed that SlHDA7 eliminates the acetyl group from the RIN and FUL1 chromatin, leading to their downregulation (Fig. 7). Because HDACs lack DNA binding ability, they are typically recruited by various transcription factors or interacting proteins to form complexes that regulate target gene expression. It is probable that SlHDA7 identifies its target genes through interactions with transcription factors or other proteins.

In summary, our results indicate that SlHDA7 might function as a suppressor of fruit maturation by deacetylating H4ac to inhibit the transcription of genes participating in ethylene synthesis and signaling, carotenoid biosynthesis, cell wall metabolism, and crucial ripening-related transcription factors in tomato, providing new insights into the impact of epigenetic regulation on fruit maturation.

## Materials and methods

### Plant materials and growth conditions

*Solanum lycopersicum* (cv. Ailsa Craig (AC)) wild-type (WT) and transgenic plants were cultivated in a greenhouse with natural light conditions (23°C with a 16 h/8 h photoperiod). The fruits’ development and ripening stages were accurately determined by tagging at anthesis, which was recorded as days post-anthesis (dpa). The pericarp of harvested fruits were promptly frozen in liquid nitrogen and preserved at −80°C for additional analysis.

### Generation of transgenic plants

The coding sequence of *SlHDA7* (Solyc01g009110) was cloned and inserted into the pBI121-GFP vector to create the pBI121-SlHDA7-GFP construct. To produce the *slhda7* mutants, two specific sgRNA sequences were inserted into the pPTG-sgRNA-Cas9-AtU6–1 vector to target SlHDA7 simultaneously [55]. The resulting vectors were confirmed through sequencing, then transformed into the cotyledon explants of the tomato cultivar AC by *Agrobacterium tumefaciens GV3101* mediated transformation [56]. Kanamycin-resistant, PCR and Sanger sequencing were used for identifying positive transformants. All primers used in this study were listed in Table S1 (see online supplementary material).

### Fruit ripening characteristics

The ethylene production rate was determined following a previously published protocol [57, 58]. Chlorophyll and carotenoids were extracted and measured as previously described [59] and are expressed as μg g^−1^ FW. The propectin and soluble pectin contents were measured as described previously [60]. Three biological replicates were conducted, with one replicate consisting of six fruits.

### RT-qPCR assays

Total RNA of roots, stems, leaves, flowers, and tomato pericarp at 10, 20, 30, 36, 39, and 40 dpa was extracted with the HiPure Plant RNA Mini Kit (Magen, Guangzhou, China). First-strand cDNA was synthesized with a PrimeScript TM Reagent Kit (RR036A, Takara Bio Inc., Shiga, Japan) following the method described previously [56]. RT-qPCR was performed with an ABI7500 Real-Time PCR System (Thermo Fisher Scientific, Waltham, MA, USA) via using SYBR® Premix Ex TaqTM II (RR420A, TakaraBio Inc., Shiga, Japan). The gene expression level was calculated using the 2^−ΔΔCt^ method. The analysis included three independent biological replicates. *ACTIN* (Solyc03g078400) was chosen as the reference control.

### RNA-seq assay

RNA was extracted from three independent WT and *slhda7* fruits at 36 dpa. The RNA extraction, library preparation, and Illumina sequencing were carried out at the Gene Denovo Company (Guangzhou, China) using the Illumina NovaSeq 6000 platform. The clean reads were compared with HISAT2 to the reference genome SL3.0. Gene expression levels were quantified using an RPKM value. Differentially expressed genes (DEGs) were identified based on the fold changes >2.5 with FDR <0.05 using DESEQ2. The gene functional enrichment analysis of the DEGs was conducted using the DAVID database (https://david.ncifcrf.gov/gene2gene.jsp).

### Analysis of global histone acetylation

The levels of global histone acetylation in the leaves were assessed following previously established protocols [61]. Specific anti-acetylated histone antibodies were utilized for Western blot analysis. The quantification of immunoblotting signals was carried out using ImageJ. The specific anti-acetylated antibodies used in western blot assay were listed as: anti-H3 (ab1791; Abcam), anti-H3ac (17–615; Millipore), anti-H3K9ac (ab32129; Abcam), anti-H3K14ac (ab52946; Abcam), anti-H4 (ab177840; Abcam), and anti-H4ac (06–866; Millipore).

### ChIP-qPCR analysis

ChIP-qPCR assays were conducted following the method described previously [13]. Fruits from the WT, *slhda7–1*, and SlHDA7–36 lines at 36 dpa were collected and promptly fixed with 1% formaldehyde under vacuum for 15 min, then quenched by adding glycine. After crosslinking, the chromatin complex was sonicated to approximately 500 bp fragments. Immunoprecipitation was carried out using an anti-acetyl-histone H4ac antibody (06–866; Millipore). The enrichment was assessed through qPCR and was normalized to the relative enrichment compared to the input. All primers utilized in ChIP-qPCR are listed in Table S1 (see online supplementary material).

### Postharvest storage test

The fruits of the WT, *SlHDA7* overexpression lines, and *SlHDA7* knockout mutants were collected at the breaking stage and placed on a clean plate under greenhouse conditions for 16 hours during the day (25°C) and 8 hours during the night (22°C). The phenotype and fruit weight were monitored every 7 days. Each postharvest storage test utilized 20 fruits.

### Statistical analysis

We used SPSS version 7.5 for statistical analysis. Variations among different sample groups were conducted with either Student’s *t*-test or ANOVA.

## Supplementary Material

Web_Material_uhae234

## Data Availability

The RNA-Seq data have been deposited in the Dryad in the following repository (https://datadryad.org/stash/share/dFeS6NvPQK_xhouVJUpnPQYRN2R5VkjHNAvbqiel2R8).
