# Supplementary material for: Histone deacetylase SlHDA7 impacts fruit ripening and shelf life in tomato
Source: Hortic Res. 2024 Aug 14;11(11):uhae234. doi: 10.1093/hr/uhae234 (PMC11534877; doi:10.1093/hr/uhae234)
Supplement: Web_Material_uhae234 [file web_material_uhae234.zip › Supplement figures.docx]

**Histone deacetylase SlHDA7 impacts fruit ripening and shelf life in tomato**

Yijie Zhou^a, b, #^, Zhiwei Li^a c, d, #^, Xinguo Su^b^, Huiyu Hou^a, c^, Yueming Jiang^a, c, d^, Xuewu Duan^a, c, d^, Hongxia Qu^a, c, d, *^, Guoxiang Jiang^a, c, d, *^

^a^ State Key Laboratory of Plant Diversity and Specialty Crops & Guangdong Provincial Key Laboratory of Applied Botany, South China Botanical Garden, Chinese Academy of Sciences, Guangzhou 510650, China

^b^ Guangdong AIB Polytechnic, Guangzhou 510507, China

^c^ South China National Botanical Garden, Guangzhou 510650, China

^d^ University of Chinese Academy of Sciences, Beijing 100049, China

**Running title**: Histone deacetylase SlHDA7 represses tomato fruit ripening

^#^These authors contribute equally to this work.

**^*^Corresponding authors:**

Guoxiang Jiang, Tel: +86 20 87578854; Email: [gxjiang@scbg.ac.cn](mailto:gxjiang@scbg.ac.cn).

Hongxia Qu, Tel: +86 20 87579147; Email: q-hxia@scbg.ac.cn

Emails address: yijiezhou@gdaib.edu.cn (Yijie Zhou); lizhiwei@scbg.ac.cn (Zhiwei Li); suxg@gdaib.edu.cn (Xinguo Su); huiyuhou@scbg.ac.cn (Huiyu Hou); [ymjiang@scbg.ac.cn (Yueming](mailto:ymjiang@scbg.ac.cn%20(Yueming) Jiang); xwduan@scbg.ac.cn (Xuewu Duan); q-hxia@scbg.ac.cn (Hongxia Qu); gxjiang@scbg.ac.cn (Guoxiang Jiang)


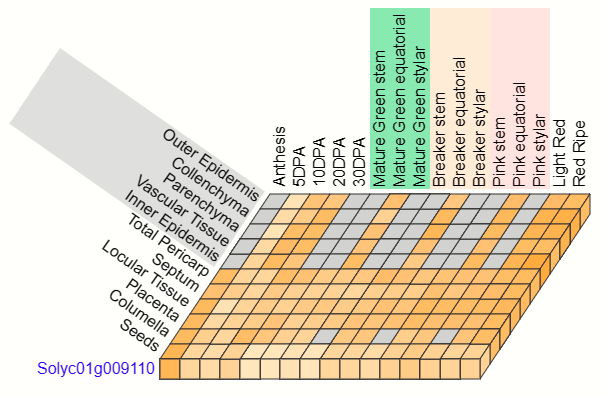


**Fig. S1** Heatmap showing the expression of SlHDA7 in various fruit tissues during different developmental stages of tomato fruit. Expression images and data for analysis were obtained from the Tomato Expression Atlas (<https://tea.solgenomics.net/>), derived from the cultivar M82.


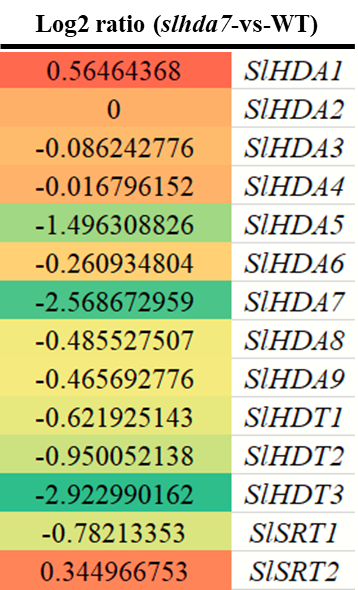


**Fig. S2** Heat map showing the expression levels of *SlHDACs* in the fruits of WT and *slhda7* mutant at 36 dpa by RNA-seq.


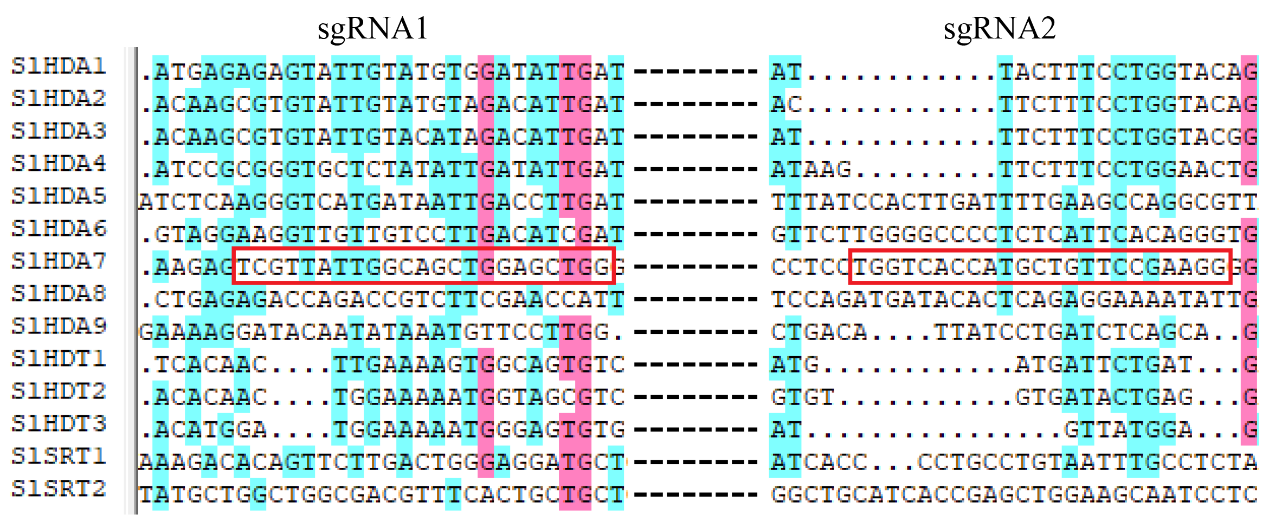


**Fig. S3.** Multiple sequence alignment of sgRNA1 and sgRNA2 among tomato *SlHDACs*.
